# Supplementary material for: HIVprotI: an integrated web based platform for prediction and design of HIV proteins inhibitors
Source: J Cheminform. 2018 Mar 9;10:12. doi: 10.1186/s13321-018-0266-y (PMC5845081; doi:10.1186/s13321-018-0266-y)
Supplement: Supplementary file 2 — Additional file 2. Source code of HIVProtI web server. [file 13321_2018_266_MOESM2_ESM.zip › HIVprotI_Source-code/hivprotI/jsmol/jsmetest2.htm]

Jmol/JME Demo


|  |  |  |  |
| --- | --- | --- | --- |
| Test of Jmol/JME interface -- switch back and forth between 2D and 3D, changing the model as desired. Status: working; 3D->2D loses stereochemistry. Requires JSmolJME.js. Code on page:  ``` jmol = Jmol.getApplet("jmol", JmolInfo) jme = Jmol.getJMEApplet("jme", JMEInfo, jmol); ``` |  |  | info clear no info |
